# Cost Effectiveness Analysis of Eribulin Mesylate as a Treatment for Metastatic Breast Cancer in Spain: Management in the Later Line of Therapy

**DOI:** 10.36469/9834

**Published:** 2015-10-21

**Authors:** Gabriel Tremblay, Unnati Majethia, Ilias Kontoudis, Jesús De Rosendo

**Affiliations:** 1 Eisai Inc., Woodcliff Lake, NJ, USA; 2 Eisai Limited, European Knowledge Centre, Mosquito Way, Hatfield, Hertfordshire, AL10 9SN; 3 Eisai Farmacêutica S.A., Madrid, Spain

**Keywords:** spain, cost-utility, cost-effectiveness, eribulin, her2-negative, metastatic breast cancer

## Abstract

**Background:** Two thirds (62%) of metastatic breast cancer (MBC) patients in Western Europe have human epidermal growth factor receptor 2 (HER2)-negative disease, for which anthracyclines and taxanes are recommended as first-line treatments, followed by microtubule-targeting agents such as capecitabine, vinorelbine and/or eribulin. The study objective was to compare the cost-effectiveness of eribulin in Spain as a second-line treatment for HER2-negative MBC with its current status as a third-line treatment for patients who have received capecitabine.

**Methods:** A Markov model was developed from the perspective of the Spanish healthcare system. The model had three health states: Stable; Progression and Death. In Stable, patients received eribulin or: capecitabine and vinorelbine for HER2-negative patients; primary treatment of physician’s choice (TPC) for post-capecitabine patients. In Progression, all patients received secondary TPC. Model inputs were overall survival, progression-free survival and costs relating to chemotherapies, grade 3/4 adverse events and healthcare utilization. Sensitivity analyses were conducted to identify uncertainty.

**Results:** As second-line treatment, Eribulin was associated with a greater incremental benefit in life years (LYs) and quality-adjusted life years (QALYs) than capecitabine and vinorelbine. Erubilin as third-line treatment was associated with greater benefit in life years (LYs) and QALYs than TPC. The incremental cost-effectiveness ratios (ICERs) for eribulin were higher in the second-line than the third-line setting in terms of LYs (€35,149 versus €24,884) and QALYs (€37,152 versus €35,484). In both settings, deterministic sensitivity analyses demonstrated that the ICER is most sensitive to the eribulin price.

**Conclusion:** Eribulin is cost-effective as second-line treatment for HER2-negative MBC patients in Spain; albeit, slightly less so than as third-line treatment for MBC patients who have received capecitabine (an ICER per QALY difference of €1,668). This difference may fall within the margin of error for the model and could potentially be addressed by a minor reduction in the eribulin price.

## BACKGROUND

Breast cancer has the highest incidence of all cancers (>1.67 million incident cases each year) and is the highest cancer-related cause of mortality in women worldwide (responsible for nearly 522,000 deaths each year).[1] In Spain, there are over 25,000 incident cases (with the highest incidence occurring in patients aged 45–65 years) and approximately 6,000 breast cancer deaths each year.[1,2]

Despite advances in diagnosis and treatment, metastatic breast cancer (MBC) remains incurable with a median survival of 2–3 years.[3,4] Approximately 62% of patients in Western Europe have MBC that is characterized by low/absent expression of human epidermal growth factor receptor 2 (HER2).[5] HER2-negative disease can be associated with a poor prognosis – particularly if hormonal receptors are also low/ absent, in which case treatment options are limited.[6]

The recommended treatment for HER2-negative MBC is single agent chemotherapy, with combination chemotherapy reserved for situations of visceral crisis, rapidly progressive or highly symptomatic disease. As a first-line treatment, anthracycline- and taxane-based chemotherapy is typically administered, followed by agents such as capecitabine (an antimetabolite)7 and vinorelbine (an anti-mitotic chemotherapy)[8] as second-line treatments.[4]

Eribulin is a non-taxane inhibitor of microtubule dynamics that has provided an overall survival (OS) benefit in heavily pre-treated patients (up to five lines of treatment in the phase III trial EMBRACE)[9] and similar progression-free survival (PFS) and OS results to capecitabine after prior treatment with an anthracycline and taxane in the phase III trial E7389-G000-301 (hereafter referred to as 'study 301').[10]

Study 301 and EMBRACE were used to demonstrate the safety and efficacy of eribulin as second- and third-line therapy, respectively. Based on these studies, eribulin was approved in Europe for the treatment of patients with locally advanced breast cancer or MBC who have progressed after at least one chemotherapy (prior therapy should have included an anthracycline and a taxane).[11] Eribulin is recommended by the Spanish Agency of Medicines and Health Products for the treatment of patients with advanced breast cancer or MBC who, in addition to having been given taxanes and anthracyclines, have also been treated with capecitabine.[2,12] The third line indication in Spain has recently been extended to a second line indication, after capecitabine. Spain, similar to many European countries, has national and regional health technology assessment (HTA) agencies and requires economic evidence for local reimbursement of drugs.

The objective of this study was to compare the cost-effectiveness of eribulin as a second-line treatment for HER2-negative MBC with its current status in Spain as a third-line treatment for patients who have progressed on/are refractory to capecitabine. This was achieved using a 5-year partitioned survival model.

## METHODOLOGY

### Model Perspective and Patient Populations

A Markov model was developed from the perspective of the Spanish healthcare system. Two pre-treated patient populations were included: HER2-negative patients eligible for second-line therapy (modelled on a subgroup of patients in study 301 [data on file]); and patients who had progressed on/were refractory to capecitabine and eligible to receive third-line therapy (modelled on a subgroup of patients in EMBRACE [data on file]; hereafter referred to as post-capecitabine patients). All patients had received an anthracycline and a taxane unless contraindicated.

## Health States and Treatments

The model had three health states: Stable; Progression and Death (Figure 1). Patients entered Stable upon initiation of primary therapy: HER2-negative patients received eribulin or capecitabine and vinorelbine; post-capecitabine patients received eribulin or treatment of physician's choice (TPC – a mixture of agents mirroring clinical practice in Spain; referred to as 'primary TPC' in Stable). On disease progression, patients entered Progression and received TPC (referred to as 'secondary TPC' in Progression) for 8 months (HER2-negative patients) or 6 months (post-capecitabine patients), on the basis that the average number of cycles of MBC treatments in Western Europe is 7.35 post-second-line and 5.83 post-third-line.[13] Patients in Stable and Progression could transition to Death.

Primary and secondary therapies were composed of the most commonly used treatments for post-secondline management of MBC in Western Europe (compositions outlined in Figure 1).[13] Treatment utilization was informed by physician input and 10% dose rounding was employed. Patients were assumed refractory to a particular treatment if their disease progressed whilst receiving that treatment and were switched to an alternative treatment, with the view to achieving stable state. A patient, who received eribulin as second-line treatment and progressed, would not receive this in subsequent lines of treatment (Figure 1, top panel). The same applied to the use of eribulin in third-line treatment (Figure 1, bottom panel). Treatment cycles lasting 21 days (based on labelling information)[7,8,11,14-16] were converted to 30.42 day Markov cycles (hereafter referred to as months).

Movement of patients between health states was based on data from the clinical study reports (CSRs) of study 301 (HER2-negative patients) and EMBRACE (post-capecitabine patients). Due to the change in treatment upon progression, a patient cannot be in stable state more than once, in any given line of therapy (as the additional stable states would be generated by a drug other than the primary drug). A unidirectional (or one-way) transition aspect was utilized to internalize this concept in a partitioned survival model.

**Figure 1.** Model Structure

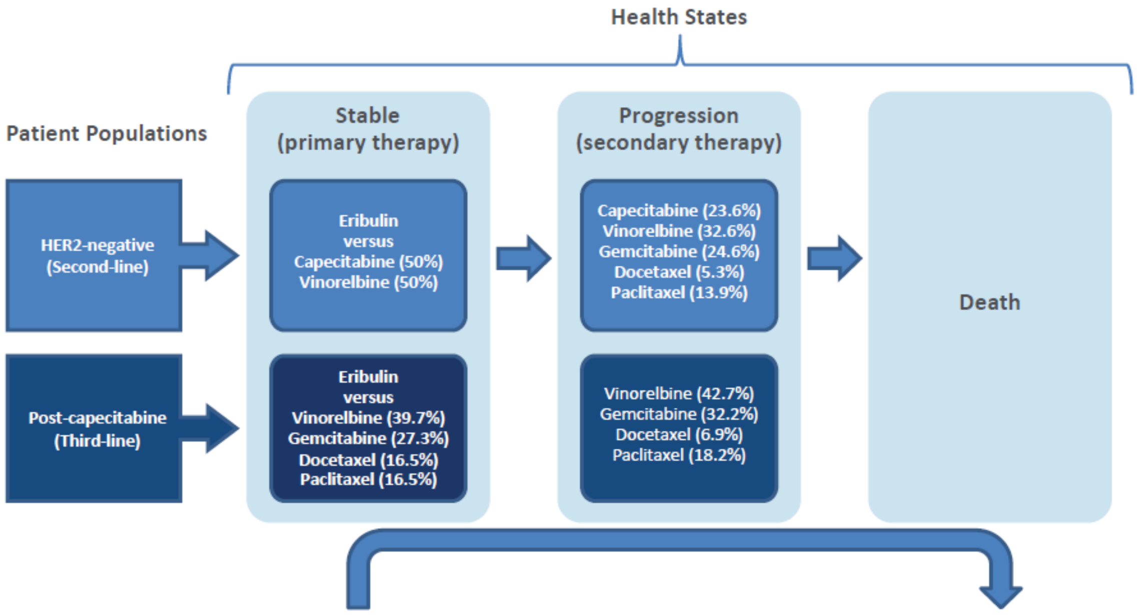

HER2: human epidermal growth factor receptor 2

## Efficacy Measures and Survival Extrapolation

The model considered OS and PFS. OS for post-capecitabine patients was extrapolated to five years to match study 301 and because 23% of post-capecitabine patients were alive at the 34-month cut-off. The conceptual framework for the extrapolation was based on that of a previously reported study.[17] After testing for the proportional hazard (PH) assumption using visual inspection and a PH global test (Schoenfeld residuals), a piecewise model using the Kaplan-Meier estimator was employed until the 34-month cut-off, with an extrapolated parametric tail up to five years. Six distribution forms (Weibull, exponential, loglogistic, log-normal, gamma and Gompertz) were evaluated and the gamma distribution was selected as having the best fit. For HER2-negative patients receiving eribulin versus capecitabine and vinorelbine, mean OS was modelled at 21.10 versus 17.98 months and mean PFS was modelled at 4.20 versus 4.17 months, respectively. For post-capecitabine patients receiving eribulin versus primary TPC, mean OS was modelled at 16.36 versus 12.64 months and mean PFS was modelled at 4.06 versus 3.80 months, respectively.

## Costs

The model considered costs relating to chemotherapies (Table 1) and adverse events (AEs; Table 2), as well as healthcare utilization costs: medical costs incurred from initiation of primary therapy until palliation; palliative care costs in the last 6 months of life; and end-of-life costs in the last 0.5 months of life (Table 3). Unit costs and treatment costs were sourced from IHS Global Insight.[18] Micro-costing for AEs was based on a previously published methodology.[19] All other direct medical costs were sourced from Duran et al. (2014) and Hechmati et al. (2011).[20,21] Costs were inflation-adjusted to 2015 using an average annual rate of 2.12% from 2010–2013 (2.99% in 2010, 2.38% in 2011, 2.87% in 2012 and 0.25% in 2013 [rates sourced from El Instituto Nacional de Estadistica]). Total costs were discounted at 3% per year according to World Health Organization (WHO) guidelines for cost-effectiveness analysis (Table 4).[22]

## Adverse Events

The model considered grade 3/4 AEs observed in ≥5% of patients from study 301 that required treatment and/or hospitalization. The incidence rate of AEs was assumed to be constant due to lack of contradictory evidence and consistency with other cost-effectiveness models. Healthcare utilization for the treatment of AEs was based on physician input.

## Utilities

The model presents life years (LYs) and quality-adjusted life years (QALYs). Utilities were derived from study 301 as health-related quality of life data were not collected in EMBRACE.[9,10] An ordinary least squares regression algorithm (previously published and validated) was employed to map QLQ-C30 scores onto the EQ-5D.[23] The resulting EQ-5D scores were used to infer utilities for the following states: Baseline; Tumor Response and Progression. To determine the utility for the stable disease state (non-progressive), the incremental utility of Tumor Response was multiplied with the tumor response rate observed in study 301, added to the Baseline score, and the annual disutility of AEs (estimated using independent linear mixed-effects models and pooled data from study 301 and EMBRACE; Table 5) was subtracted (Table 6).[24,25] Benefits were discounted at 3% per year according to World Health Organization (WHO) guidelines for cost-effectiveness analysis.[22]

**Table 1.** Dosing Information and Average Costs for Chemotherapies

| Drug | ROA | Dose ($mg/m^2$) | Doses per cycle | Unit Strength (mg) | Pack Size | Unit Cost[18] | Drug Cost per Dose[¥] | Admin. Cost per Dose[†20,21] | Drug Cost PPPM | Admin. Cost PPPM |
|---|---|---|---|---|---|---|---|---|---|---|
| Eribulin[11] | IV | 1.23 | 2 | 0.88 | 1 | € 320 | € 656 | € 213 | € 1899 | € 618 |
| | | | | 1.32 | 1 | € 480 | | | | |
| Capecitabine[7] | Oral | 2500 | 14 | 150 | 60 | € 0.35 | € 8 | € 63 | € 170 | € 92 |
| | | | | 500 | 120 | €1.15 | | | | |
| Vinorelbine[8] | Oral | 75 | 2 | 20 | 1 | € 45.20 | € 249 | € 63 | € 721 | € 92 |
| | | | | 30 | 1 | € 67.80 | | | | |
| Gemcitabine[14] | IV | 1250 | 2 | 200 | 1 | € 8.74 | € 80 | € 213 | € 231 | € 618 |
| | | | | 1000 | 1 | € 43.70 | | | | |
| | | | | 2000 | 1 | € 87.40 | | | | |
| Docetaxel1[5] | IV | 100 | 1 | 20 | 1 | € 43.97 | € 322 | € 213 | € 467 | € 309 |
| | | | | 80 | 1 | € 175.88 | | | | |
| | | | | 160 | 1 | € 351.76 | | | | |
| Paclitaxel[16] | IV | 175 | 1 | 30 | 1 | € 29.55 | € 294 | € 213 | € 426 | € 309 |
| | | | | 100 | 1 | € 101.95 | | | | |
| | | | | 150 | 1 | € 175.88 | | | | |
| | | | | 300 | 1 | € 392.67 | | | | |
| **Primary eribulin costs** | | | | | | | | | **€ 1899** | **€ 618** |
| **Primary capecitabine & vinorelbine costs** | | | | | | | | | **€ 445** | **€ 92** |
| **Primary TPC costs** | | | | | | | | | **€ 497** | **€ 307** |
| **Secondary TPC (HER2-negative patients) costs** | | | | | | | | | **€ 416** | **€ 263** |
| **Secondary TPC (post-capecitabine patients) costs** | | | | | | | | | **€ 492** | **€ 316** |

Based on a mean body surface area of $1.74 \pm 0.19 m^2$ and a dose intensity of 0.84; Admin: administration; IV: intravenous; ROA: route of administration; TPC: treatment of physician's choice

[†]Administration costs consisted of costs for laboratory tests (€63 per patient) plus an additional cost for 2 hours of nurse care for IV chemotherapies (€150 per patient).

**Table 2.** Monthly Per Patient Costs Attributed to Grade 3/4 Adverse Events

| | | Eribulin | | | Primary Comparator | | | Secondary TPC | | |
|---|---|---|---|---|---|---|---|---|---|---|
| AE | Tx Cost for AEs[18] | Tx Rate | Hosp. Rate | Average Cost* | Tx rate | Hosp. Rate | Average Cost* | Tx Rate | Hosp. Rate | Average Cost* |
| Neutropenia | € 120 | 3.32% | 0.23% | € 8.10 | 0.51% | 0.05% | € 1.46 | 1.59% | 0.00% | € 1.92 |
| FN | € 125 | 0.65% | 0.59% | € 11.40 | 0.23% | 0.19% | € 3.66 | 0.50% | 0.25% | € 5.11 |
| Leukopenia | € 177 | 1.18% | 0.00% | € 2.10 | 0.28% | 0.00% | € 0.50 | 0.50% | 0.00% | € 0.88 |
| Diarrhea | € 1 | 0.11% | 0.02% | € 0.41 | 1.29% | 0.56% | € 10.07 | 0.00% | 0.00% | € 0.00 |
| Asthenia/fatigue | € 2 | 0.29% | 0.18% | € 3.28 | 0.19% | 0.33% | € 5.89 | 0.25% | 0.37% | € 6.73 |
| PN | € 11 | 0.74% | 0.09% | € 1.72 | 0.00% | 0.00% | € 0.00 | 0.12% | 0.00% | € 0.01 |
| Dyspnea | € 1 | 0.54% | 0.27% | € 4.91 | 0.74% | 0.56% | € 10.07 | 0.99% | 0.74% | € 13.40 |
| PPE | € 5 | 0.02% | 0.02% | € 0.41 | 1.42% | 0.09% | € 1.77 | 0.62% | 0.12% | € 2.28 |
| Total | | | | € 32.33 | | | € 33.42 | | | € 30.33 |

*Hospitalizations for adverse events were assumed to cost €1804 per patient (€269.26 per day for 6.7 days)[20,21]

AE: adverse event; FN: febrile neutropenia; hosp: hospitalization; PN: peripheral neuropathy; PPE: palmar-plantar erythrodysesthesia; TPC: treatment of physician's choice; Tx: treatment

**Table 3.** Healthcare Utilization Costs per Patient per Month[20,21]

| Cost Type | Components |
|---|---|
| Medical | Oncologist follow-up |
| Palliative Care | Oncologist follow-up |
| | Moderate pain relief with co-codamol 8/500 |
| | Bone pain relief with ibandronate |
| | Constipation relief with senna |
| | Inflammation relief with dexamethasone |
| | Laboratory tests* |
| End-of-life | Hospitalization (applicable for 40% of patients) |
| | Hospice care (applicable for 10% of patients) |
| | Community support at home (two visits per day; applicable for 50% of patients) |

*Laboratory tests included complete blood counts, blood chemistry panels, liver function tests, and tests for urea and electrolytes

**Table 4.** Total Discounted Costs[18,20,21]

| Cost Type | Component Costs | HER2-negative Patients | | | Post-capecitabine Patients | | |
|---|---|---|---|---|---|---|---|
| | | Eribulin | Capecitabine & vinorelbine | Difference | Eribulin | Primary TPC | Difference |
| **Drugs** | Primary therapy | € 7858 | € 1,831 | € 6027 | € 7619 | € 1859 | € 5760 |
| | Secondary therapy | € 1561 | € 1459 | € 102 | € 1168 | € 1104 | € 64 |
| | Drug administration | € 3544 | € 1298 | € 2246 | € 2629 | € 1531 | € 1098 |
| **Direct Medical** | Medical | € 1315 | € 1074 | € 241 | € 902 | € 634 | € 268 |
| | Palliative care | € 737 | € 715 | € 21 | € 817 | € 746 | € 71 |
| | End-of-life | € 3765 | € 3916 | -€ 151 | € 183 | € 188 | (€ 5) |
| **AEs** | Grade 3/4 AEs | € 620 | € 536 | € 84 | € 202 | € 193 | € 9 |
| **Total** | | € 19 400 | € 10 830 | € 8570 | € 13 519 | € 6255 | € 7264 |

AE: adverse event; HER2: human epidermal growth factor receptor 2

**Table 5.** Annual Disutility Scores for Adverse Events

| | | Incidence of grade 3/4 AEs | | | Disutility | | |
|---|---|---|---|---|---|---|---|
| **AE** | **Disutility** | **Eribulin** | **Capecitabine & vinorelbine** | **Primary TPC** | **Eribulin** | **Capecitabine & vinorelbine** | **Primary TPC** |
| Neutropenia | -0.007 | 45.70% | 4.90% | 21.10% | -0.003 | 0.000 | -0.001 |
| FN | -0.012 | 2.10% | 0.90% | 1.20% | 0.000 | 0.000 | 0.000 |
| Alopecia | 0.000 | 0.00% | 0.00% | 9.70% | 0.000 | 0.000 | 0.000 |
| Leukopenia | -0.003 | 15.10% | 2.00% | 5.70% | 0.000 | 0.000 | 0.000 |
| Diarrhea | -0.006 | 1.10% | 5.30% | 0.00% | 0.000 | 0.000 | 0.000 |
| Asthenia/fatigue | -0.029 | 6.20% | 6.10% | 10.10% | -0.002 | -0.002 | -0.003 |
| PN | -0.014 | 3.50% | 0.50% | 2.90% | 0.000 | 0.000 | 0.000 |
| Dyspnea | -0.027 | 2.20% | 3.80% | 2.80% | -0.001 | -0.001 | -0.001 |
| PPE | 0.000 | 0.00% | 14.50% | 3.60% | 0.000 | 0.000 | 0.000 |
| Total disutility | | | | | -0.007 | -0.004 | -0.006 |

AE: adverse event; FN: febrile neutropenia; PN: peripheral neuropathy; PPE: palmar-plantar erythrodysesthesia; TPC: treatment of physician's choice

**Table 6.** Utility Scores

| Health State | Eribulin | Capecitabine and vinorelbine | Primary TPC |
|---|---|---|---|
| **Non-progression** | 0.717 | 0.692 | 0.712 |
| Baseline | 0.713 | 0.688 | 0.713 |
| Incremental utility of tumor response | 0.088 | 0.120 | 0.088 |
| Tumor response rate | 12% | 6% | 6% |
| Disutility of AEs | -0.0069 | -0.0037 | -0.0059 |
| **Progression** | 0.695 | 0.647 | 0.695 |

AE: adverse event; PFS: progression-free survival; TPC: treatment of physician's choice

### Sensitivity Analyses

Sensitivity analyses were conducted to identify uncertainty in the results of the cost-effectiveness analysis (as recommended by the National Institute for Health and Care Excellence).[26] Deterministic sensitivity analyses were conducted to evaluate variables that were not evaluated directly in study 301 or EMBRACE (Table 7). The sensitivity of utilities and survival variables were analyzed in a probabilistic sensitivity analysis (PSA; Table 8). Monte Carlo simulations were repeated over 1000 iterations to create an incremental costeffectiveness plane and a cost-effectiveness acceptability curve to illustrate the probability of achieving cost-effectiveness given a range of ICER per QALY thresholds.

**Table 7.** Scenario Presentations

| Scenario Presentation | Optimistic | Base Case | Conservative |
|---|---|---|---|
| Scenario 1: Benefits discounting rate | 0.0% | 3% | 5.0% |
| Scenario 2: Costs discounting rate | 5.0% | 3% | 0.0% |
| Scenario 3: Costs and benefits discounting rates | 0.0% | 3% | 5.0% |
| Scenario 4: Eribulin price | -35.0% | 0.0% | 20.0% |
| Scenario 5: Comparator price | 20.0% | 0.0% | -20.0% |
| Scenario 6: Administration costs | -20.0% | 0.0% | 20.0% |
| Scenario 7: Direct healthcare costs | -20.0% | 0.0% | 20.0% |

**Table 8.** Parameters

| | | | HER2-negative patients Post-capecitabine Patients | | Post-capecitabine Patients | | | |
|---|---|---|---|---|---|---|---|---|
| **Variable** | **Intervention** | **Parameter** | **Point Estimate** | **SE** | **Point Estimate** | **SE** | **Distribution** | **Justification** |
| Utility | Eribulin | Baseline | 0.713 | 0.23 | 0.713 | 0.23 | Gamma | Study 301 data |
| | | Tumor response | 0.801 | 0.19 | 0.801 | 0.19 | Gamma | Study 301 data |
| | | Progression | 0.695 | 0.21 | 0.695 | 0.21 | Gamma | Study 301 data |
| | Comparator | Baseline | 0.688 | 0.23 | 0.713 | 0.23 | Gamma | Study 301 data |
| | | Tumor response | 0.808 | 0.19 | 0.801 | 0.19 | Gamma | Study 301 data |
| | | Progression | 0.647 | 0.21 | 0.695 | 0.21 | Gamma | Study 301 data |
| Survival* | Eribulin | Pre-progression | 4.20 | 0.23 | 4.06 | 0.2 | Normal | Mean from parametric simulation SD from trial data |
| | | Post-progression | 16.90 | 0.56 | 12.29 | 0.56 | Normal | Mean from parametric simulation SD from trial data |
| | Comparator | Pre-progression | 4.17 | 0.2 | 3.8 | 0.23 | Normal | Mean from parametric simulation SD from trial data |
| | | Post-progression | 13.81 | 0.74 | 8.85 | 0.74 | Normal | Mean from parametric simulation SD from trial data |

*End-of-life was fixed at 1 month
N/A: not applicable; SD: standard deviation; SE: standard error; TPC: treatment of physician's choice

## RESULTS

As a second-line treatment for HER2-negative patients, eribulin was associated with a greater incremental benefit in LYs (1.68 versus 1.44) and QALYs (1.18 versus 0.95) than capecitabine and vinorelbine. It was also associated with greater drug costs (€12,963 versus €4,589), direct medical costs (€5,817 versus €5,706) and AE costs (€620 versus €536) than capecitabine and vinorelbine. Total costs were €19,400 for eribulin versus €10,830 for capecitabine and vinorelbine. The ICER for eribulin compared to capecitabine and vinorelbine was €35,149 per LY and €37,152 per QALY (Table 9).

As a third-line treatment for post-capecitabine patients, eribulin was associated with a greater benefit in LYs (1.32 versus 1.02) and QALYs (0.92 versus 0.72) than primary TPC. It was also associated with greater drug costs (€11,415 versus €4,494), direct medical costs (€1,902 versus €1,569) and AE costs (€202 versus €193) than primary TPC. Total costs were €13,519 for eribulin versus €6,255 for primary TPC. The ICER for eribulin compared to primary TPC was €24,884 per LY and €35,484 per QALY (Table 9).

**Table 9.** Summary of Cost-effectiveness Results

| | HER2-negative Patients | | Post-capecitabine Patients | |
|---|---|---|---|---|
| | **Eribulin** | **Capecitabine & Vinorelbine** | **Eribulin** | **Primary TPC** |
| Incremental benefit in LYs | 1.68 | 1.44 | 1.32 | 1.02 |
| Incremental benefit in QALYs | 1.18 | 0.95 | 0.92 | 0.72 |
| Incremental drug costs | €12 963 | €4589 | €11 415 | €4494 |
| Direct medical costs | €5817 | €5706 | €1902 | €1569 |
| AE costs | €620 | €536 | €202 | €193 |
| Total incremental costs | €19 400 | €10 830 | €13 519 | €6255 |
| ICER per LY | €35 149 | | €24 884 | |
| ICER per QALY | €37 152 | | €35 484 | |

AE: adverse event; ICER: incremental cost-effectiveness ratio; HER2: human epidermal growth factor receptor 2; LY: life year; QALY: quality-adjusted life year; TPC: treatment of physician's choice

### Deterministic Sensitivity Analysis

In the second-line setting, the scenarios with the greatest effect on the ICER were the price of eribulin (€25,228–€43,965), administration costs (€35,205–€39,099) and the benefits discounting rate (€35,078–€38,534; Figure 2).

In the third-line setting, the scenarios with the greatest effect on the ICER were the price of eribulin (€22,532–€43,071), the price of the comparator (€33,780–€37,424) and the benefits discounting rate (€33,599–€36,938; Figure 3).

**Figure 2.** Tornado Graph of Deterministic Sensitivity Analysis Results (ICER per QALY) for HER2-Negative Patients receiving Second-line Therapy (€)

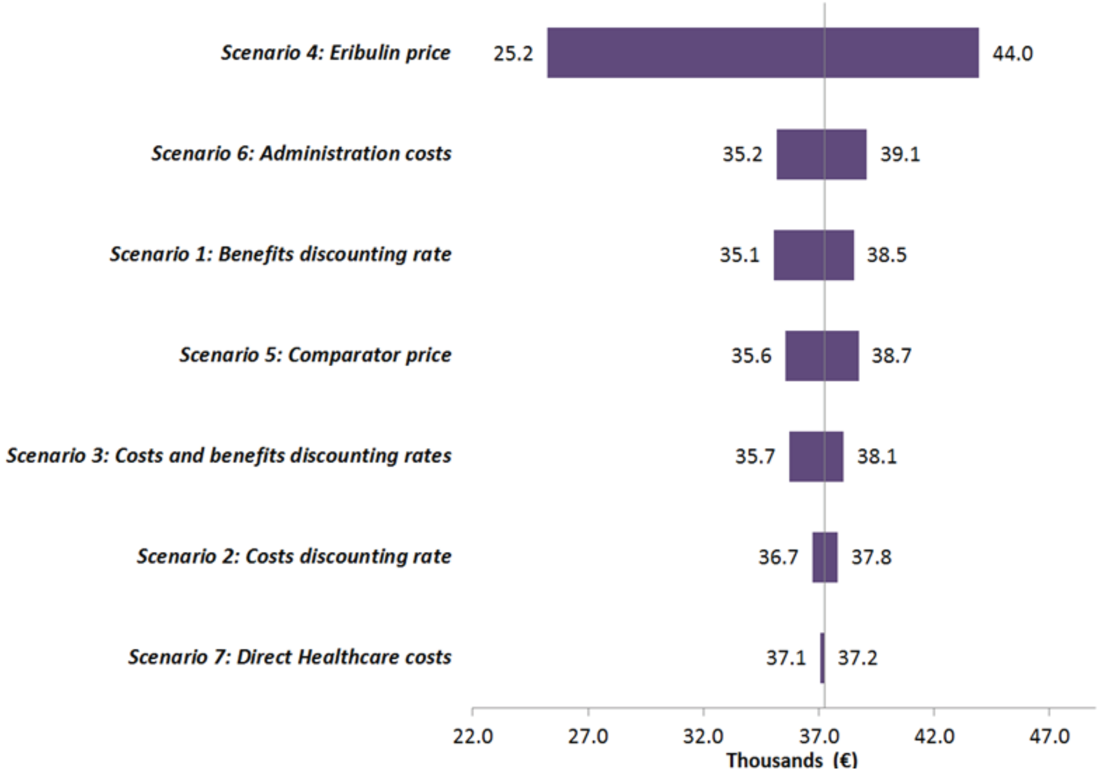

**Figure 3.** Tornado Graph of Deterministic Sensitivity Analysis Results (ICER per QALY) for Postcapecitabine Patients receiving Third-line Therapy

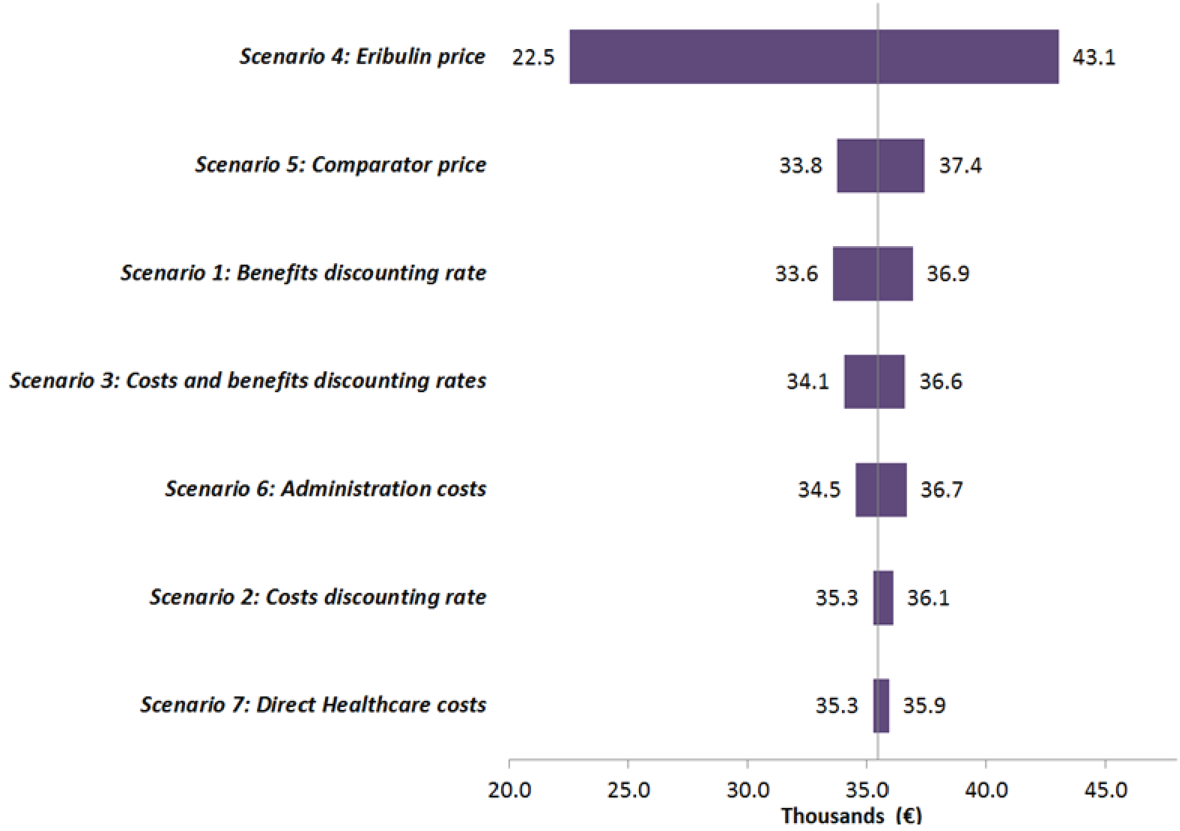

## Probabilistic Sensitivity Analysis

In the second-line setting, total incremental costs (discounted) were higher for eribulin than capecitabine and vinorelbine (€18,211 versus €9,849). The ICER for eribulin compared to capecitabine and vinorelbine was €31,914 per LY and €36,316 per QALY. In the third-line setting, total incremental costs (discounted) were higher for eribulin than capecitabine and vinorelbine (€16,413 versus €9,206). The ICER for eribulin compared to primary TPC was €23,190 per LY and €34,603 per QALY.

All PSA simulations resulted in additional QALY benefits and additional incremental costs for eribulin in both treatment settings (second-line for HER2 patients and third-line for post-capecitabine patients; Figure 4). In the second-line setting, eribulin had a 31% probability of having an ICER per QALY below €30,000 and an 81% probability of having an ICER per QALY below €45,000 (thresholds selected based on De Cock et al. [2007]). In the third-line setting, these probabilities were 37% and 73%, respectively (Figure 5).

**Figure 4.** Cost-effectiveness Plane for Eribulin

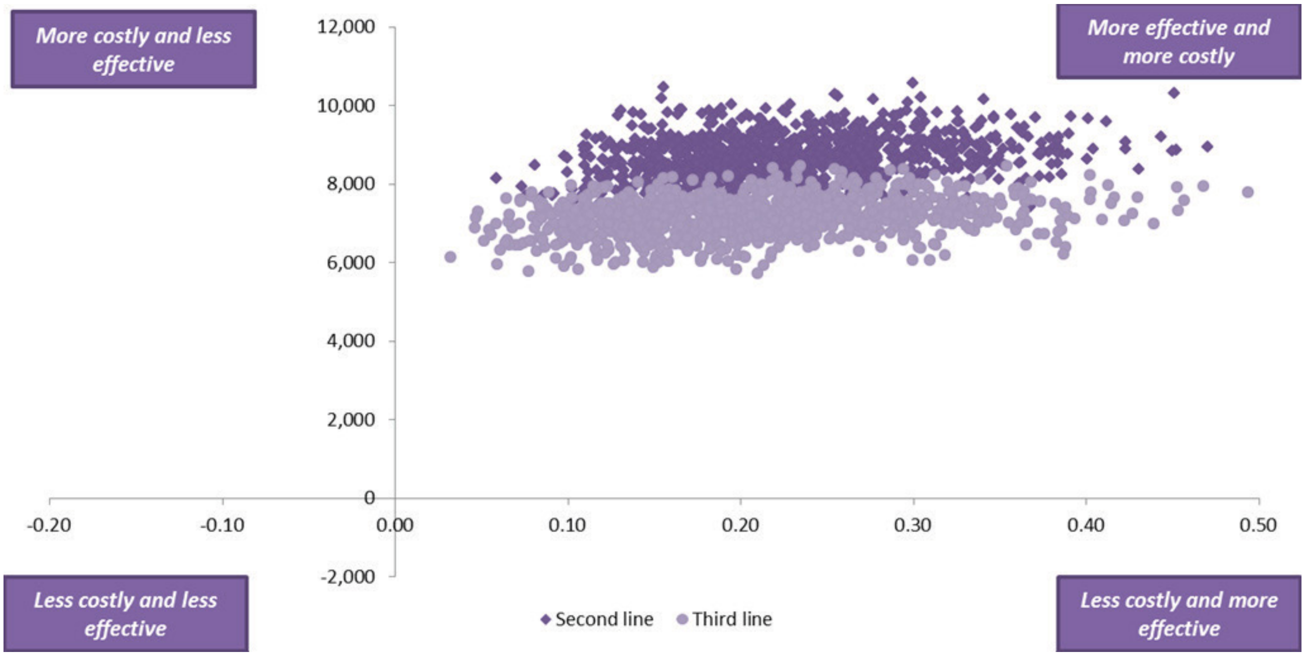

**Figure 5.** Cost-effectiveness Acceptability Curve for Eribulin

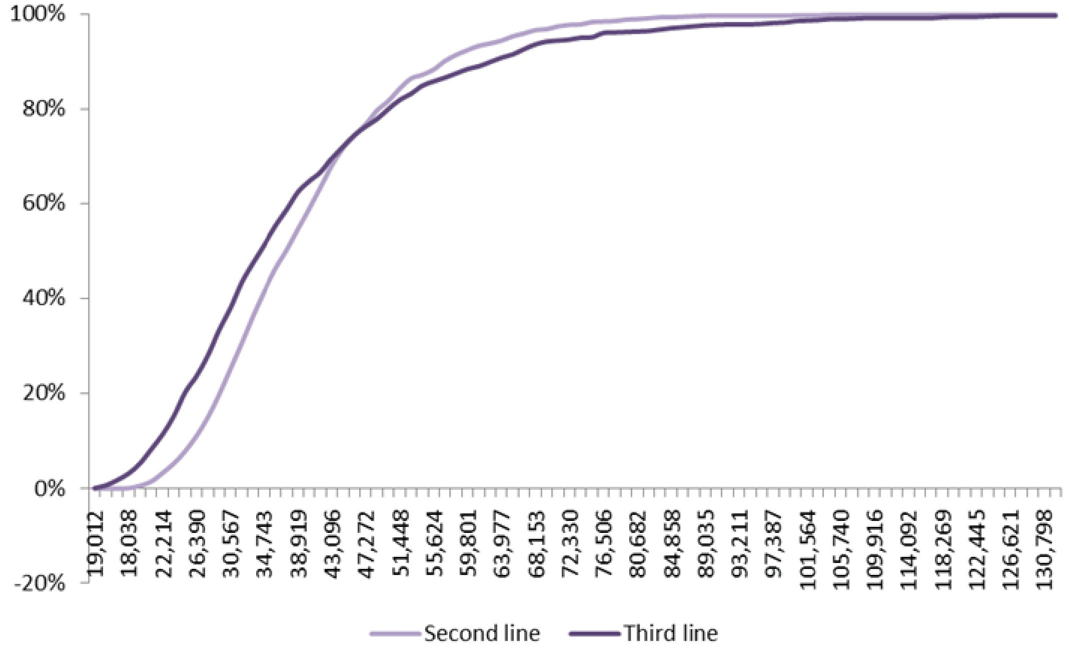

## DISCUSSION

Relative to its comparator, eribulin was associated with a lower incremental benefit in LYs (0.24 versus 0.29), a greater incremental benefit in QALYs (0.23 versus 0.20) and a greater total incremental cost (€8,570 versus €7,264) as a second-line therapy for HER2-negative patients than as a third-line therapy for postcapecitabine patients. The ICER for eribulin compared with capecitabine and vinorelbine was higher than the ICER for eribulin compared with primary TPC in terms of LYs (€35,149 versus €24,884) and QALYs (€37,152 versus €35,484).

In Spain, there is no explicitly stated threshold that defines a point at which a treatment is deemed to be cost-effective. However, an ICER between €30,000 and €45,000 is reportedly reasonable.[27] For eribulin, both ICERs fall within this range, suggesting that eribulin is a cost-effective treatment in both settings.

The ICER is €1,668 higher for eribulin in the second-line setting than the third-line setting. This indicates that eribulin is slightly less cost-effective as a second-line treatment for HER2-negative patients than it is as a third-line treatment for post-capecitabine patients. However, it is worth noting that this difference is very small – so much so that it may fall within the margin of error for the model. As such, the cost-effectiveness of eribulin may be equivalent in both treatment settings.

In both settings, the deterministic sensitivity analysis demonstrated that the ICER is most sensitive to the price of eribulin. This is illustrated by the fact that the ICER range for the eribulin price scenario was 4.8- fold and 5.6-fold greater than the range for the next most sensitive scenario in each setting; administration costs (second-line) and price of the comparator (third-line), respectively. Furthermore, 64% and 63% of the ICER ranges for the eribulin price scenario in the second-line and third-line settings, respectively, corresponded to lower ICERs. As such, a minor reduction in the price of eribulin would considerably enhance its cost-effectiveness in both treatment settings.

Total discounted costs were greater in the second-line setting than the third-line setting (€8,362 versus €7,207). The ICER for eribulin in the PSA was higher in the second-line setting than the third-line setting in terms of LYs (€31,914 versus €23,190 per LY) and QALYs (€36,316 versus €34,603 per QALY). All PSA simulations indicated that eribulin is more effective and more costly in a second-line setting. In terms of ICER thresholds, a large majority of scenarios were cost-effective at €45,000; however, a small reduction in the price of eribulin (or a larger reduction in administration costs) could be expected to significantly increase the number of cost-effective scenarios.

In terms of limitations, patient populations were based on subgroups from international trials. As the subgroups were of finite size and consisted of patients from a range of geographies, there is potential that the patient data may not to be truly reflective of Spanish MBC patients.[9,10] Model costs were adjusted from 2010 to 2015 using average inflation rates, and as such, may not have been truly reflective of present costs. Furthermore, the model did not include indirect medical or societal costs, despite the fact that MBC is associated with a substantial indirect medical cost.[28] This may have reduced the accuracy of the model outputs.

Treatment use and healthcare utilization were informed by physician input. This is a potential limitation as physician input is difficult to substantiate and may not truly reflect clinical practice. Specifically in Spain, there is a high degree of regional heterogeneity regarding pharmaceutical expenditure and prescription rates, attributed to fragmentation of pharmaceutical regulation among national and regional authorities.[29] Utilities data were based solely on study 301 via mapping QLQ-C30 scores onto the EQ-5D. As a result, utilities for post-capecitabine patients may not have been reflective of their real-life values.

To our knowledge, this is the first comprehensive attempt to compare the cost-effectiveness of eribulin as a second-line treatment for HER2-negative MBC with its current status as a third-line treatment for patients who have progressed on/are refractory to capecitabine in Spain. This is an area of interest to physicians and healthcare decision-makers who are seeking to determine the best available treatment options for MBC patients. The results of our analyses indicate that eribulin is cost-effective as a second-line treatment for HER2-negative MBC patients in Spain; albeit, slightly less so than it is as a third-line treatment for MBP patients who have progressed on/are refractory to capecitabine.

**Acknowledgements**

Editorial support was provided by Adelphi Values.

**Conflicts of Interest Declaration**

This study was funded by Eisai Inc. Eisai developed the analysis with validation of external consultants.
